# Dendrimers Target the Ischemic Lesion in Rodent and Primate Models of Nonarteritic Anterior Ischemic Optic Neuropathy

**DOI:** 10.1371/journal.pone.0154437

**Published:** 2016-04-29

**Authors:** Yan Guo, Mary A. Johnson, Zara Mehrabian, Manoj K. Mishra, Rangaramanujam Kannan, Neil R. Miller, Steven L. Bernstein

**Affiliations:** 1 Department of Ophthalmology and Visual Sciences, University of Maryland School of Medicine, Baltimore, MD, 21201, United States of America; 2 Center for Nanomedicine, Department of Ophthalmology, Wilmer Eye Institute, Johns Hopkins School of Medicine, Baltimore, MD, 21287, United States of America; 3 Division of Neuro-Ophthalmology, Department of Ophthalmology, Wilmer Eye Institute, Johns Hopkins School of Medicine, Baltimore, MD, 21287, United States of America; 4 Department of Anatomy and Neurobiology, University of Maryland School of Medicine, Baltimore, MD, 21201, United States of America; Hanson Institute, AUSTRALIA

## Abstract

**Introduction:**

Polyamidoamine dendrimer nanoparticles (~ 4 nanometers) are inert polymers that can be linked to biologically active compounds. These dendrimers selectively target and accumulate in inflammatory cells upon systemic administration. Dendrimer-linked compounds enable sustained release of therapeutic compounds directly at the site of damage. The purpose of this study was to determine if dendrimers can be used to target the optic nerve (ON) ischemic lesion in our rodent and nonhuman primate models of nonarteritic anterior ischemic optic neuropathy (NAION), a disease affecting >10,000 individuals in the US annually, and for which there currently is no effective treatment.

**Methods:**

NAION was induced in male Long-Evans rats (rNAION) and in one adult male rhesus monkey (pNAION) using previously described procedures. Dendrimers were covalently linked to near-infrared cyanine-5 fluorescent dye (D-Cy5) and injected both intravitreally and systemically (in the rats) or just systemically (in the monkey) to evaluate D-Cy5 tissue accumulation in the eye and optic nerve following induction of NAION.

**Results:**

Following NAION induction, Cy-5 dendrimers selectively accumulated in astrocytes and circulating macrophages. Systemic dendrimer administration provided the best penetration of the ON lesion site when injected shortly after induction. Systemic administration 1 day post-induction in the pNAION model gave localization similar to that seen in the rats.

**Conclusions:**

Dendrimers selectively target the ischemic ON lesion after induction of both rNAION and pNAION. Systemic nanoparticle-linked therapeutics thus may provide a powerful, targeted and safe approach to NAION treatment by providing sustained and focused treatment of the cells directly affected by ischemia.

## Introduction

Nonarteritic anterior ischemic optic neuropathy (NAION) is the most common cause of sudden optic nerve (ON)-related vision loss, annually affecting >10,000 individuals in the United States over age 50[1,2]. Although NAION’s triggering event is poorly defined[3], it is believed that the resulting vascular disruption results in a compartment syndrome in the anterior region of the optic nerve, causing progressive localized swelling[4], an inflammatory response and loss of axon integrity[5]. The combination of ischemia and inflammation results in subsequent death of retinal ganglion cells (RGCs) and varying degrees of permanent vision loss[6]. There currently is no consistently beneficial treatment for NAION[7], and the treatments that have been suggested, such as systemic corticosteroids[8], have major potential side effects. These side effects might be minimized or eliminated with selective targeting of the area of ON ischemia.

Both rodent and nonhuman primate models of nonarteritic anterior ischemic optic neuropathy (rNAION, pNAION) are similar to the human condition in many respects[9,10]. Specifically, both rNAION and pNAION induction result in vascular leakage and swelling in the anterior ON region[11], leading to progressive ON ischemia, similar to that seen in human NAION. The associated inflammation is initially acellular[12], affecting the vascular endothelium and astrocytes with release of inflammatory cytokines and breakdown of the blood-brain barrier (BBB)[11]. In the rNAION model, an inflammatory cellular invasion, comprised of extrinsic macrophages and microglia, occurs by 2 days post induction[12]. Resolution of swelling, presumably after BBB reconstitution, occurs by 5–7 days post-induction, although a cellular inflammatory infiltrate persists. The stereotyped pattern of acellular inflammatory response and BBB disruption, followed by a cellular inflammatory infiltrate is consistent with ischemic lesions seen in other locations in the CNS[13].

We have shown that intravitreal (IVT) administration of drugs that reduce ON swelling and inflammation can enable partial recovery from the ON insult and improve RGC survival in both rodent and nonhuman primate models of NAION[14,15]. However, alternative therapeutic approaches might reduce damage further, particularly if they specifically targeted the ischemic lesion.

Polyamidoamine (PAMAM) dendrimer nanoparticles (4nm) are non-biodegradable, biocompatible molecules with a tree-like (dendritic) architecture that have been explored extensively as drug and gene delivery vehicles[16]. Systemically injected dendrimers have been demonstrated to be taken up selectively by impaired CNS regions after either ischemia or trauma and to accumulate in both activated macrophages/microglia and astrocytes[17,18]. Although unmodified dendrimers are biologically inactive, PAMAM dendrimers can be linked via ester and disulfide bridges to various bioactive molecules, thus allowing high concentrations of drug to reach a specific target without producing systemic side effects, for eventual tailored release at the target cells[19]. PAMAM dendrimers also can be engineered to provide sustained release of their biological payloads within the cells of the targeted region[19,20], potentially enhancing the neuroprotective effects of the agent compared with those of free drug. We evaluated whether or not these dendrimers can target the injured cells in both our rNAION and pNAION models, and how the administration route (local via IVT injection vs. systemic via intravenous [IV] injection) affects the targeting, to determine if a dendrimer-based delivery strategy in general might be an appropriate approach to NAION treatment.

## Materials and Methods

### Animals

This study was carried out in strict accordance with the recommendations in the Guide for the Care and Use of Laboratory Animals of the National Institutes of Health. All animal protocols were approved by the University of Maryland Baltimore Institutional Animal Care and Use Committee, and all animals were handled in accordance with the ARVO Statement for the Use of Animals in Ophthalmic and Vision Research. For the murine experiments, twelve male Long-Evans rats (200-250g) were obtained from Harlan Laboratories (Indianapolis, IN). For the nonhuman primate experiment, we used an adult male rhesus monkey (*Maccaca mulatta*, age 4 years, 5kg), obtained through UMB interdepartmental transfer purchase from Dr. T. MacVittie, Department of Radiation Oncology, UMB. Permission for the nonhuman primate experiment was obtained through a separate IACUC protocol addendum approved by the whole IACUC committee.

rNAION and pNAION were induced as previously described[9,10]. Rats as well as the monkey were anesthetized using a mixture of ketamine (80mg/kg for rats, 10mg/kg for monkey) and xylazine (4mg/kg for rats, 2mg/kg for monkey). Eyes were dilated using a mixture of 1% tropicamide and 2.5% neosynephrine prior to NAION induction, IVT injections, and subsequent clinical assessments. To induce rNAION, we used an IV injection of a 2.5mM concentration of rose Bengal (90% pure: Sigma-Aldrich, St. Louis MO) in normal saline at a dose of 1ml/kg. A custom plano-convex contact lens was placed on the eye and the optic disk illuminated with 532nm laser light (500μm spot size) for 12 seconds. Eyes used for IVT injection were topically anesthetized with 0.5% proparacaine. The eyelids were scrubbed with 5% povidone iodine, and a drop of 5% povidone iodine was instilled over the eye prior to injection. IVT injections were performed using a sterile 33-gauge needle mounted on a Hamilton gas-tight microliter syringe. In all animals, rNAION was induced in one eye with the contralateral eye used as a control (see below). pNAION induction was performed in a similar fashion, but with the following modifications: 1) The concentration of rose Bengal was 2mg/ml. 2) We used a Glasser contact lens instead of a custom-designed lens. 3) The 532nm laser exposure was modified to 200mW/8.5 seconds/1.2mM spot size[15]. 4) Ofloxacin ophthalmic drops were administered prior to, and after induction. 5) Post-induction, the treated eye was covered with ophthalmic triple antibiotic ointment with 0.1% decadron (Alcon). These exposure parameters result in severe pNAION. Similar to clinical NAION, which is non-painful in presentation, the induction of rNAION and pNAION does not cause pain, which is a powerful further advantage of these models. The monkey was housed in a separate cage after induction, fed monkey chow supplemented with fruit and vegetables ad libitum, and was supplied with environmental enrichment toys as per the parent protocol.

### Targeting Studies

We used 4^th^ generation hydroxyl functionalized PAMAM-OH dendrimers (~ 14.2 kDa), in which 1–2 of the terminal hydroxyl groups were covalently-linked to the near-infrared (IR) Cy5 dye, using a stable amide linkage (D-Cy5), for both IVT and IV experiments. This conjugated dendrimer corresponds to a Cy5 payload of ~5% by weight, and was characterized extensively using 1H NMR, and HPLC [18]. The hydroxyl dendrimer was chosen because of its superior safety profile in preclinical studies, compared to amine terminated PAMAM dendrimers [17]. The detailed synthesis and the in vivo stability of this conjugate have been validated previously [18,21]. The use of a near-IR dye enabled us to minimize tissue autofluorescence, and enabled direct tissue localization analysis after early treatment and at times distant (>21 days) from rNAION induction. The D-Cy5 conjugate was stable over 72 hour period in PBS, without releasing the covalently linked Cy5[18]. The D-Cy5 extracted from the tissue and the urine in previous in vivo experiments was found to be intact over 24–48 hour period, releasing less than 5% of the conjugated Cy5[18], suggesting that the conjugate is stable in tissue. All rats (n = 12) received one dose of fluorescent dendrimer, and both retina and ON tissues were analyzed. For IVT targeting, three rats in which rNAION had been induced in one eye (n = 1) or both eyes (n = 2) were injected with a single dose of 3μl of 20mg/ml D-Cy5 solution (total 60μg) either immediately after induction (n = 1) or 1 day after induction (n = 2), and tissues were analyzed at 1 (n = 1), 2 (n = 1) or 7 days (n = 1) post-injection (Table 1). All animals were monitored daily after induction and administration. There was no observable distress requiring additional analgesics. There were no unexpected deaths.

**Table 1:** Protocol for intravitreal injections of D-Cy5 after induction.

Animal #	Eye Induced	Eye Injected	When injected**	When euthanized***
137	Right*	Right	1 day	2 days
138	Right	Both	Immediately	1 day
139	Right	Both	1 day	7 days

*Left eye was neither induced nor injected

**Time between induction and intravitreal injection.

***Time between induction and euthanasia.

For systemic targeting, nine animals had rNAION induced in one eye. Three animals were injected with a single IV dose of 20mg/kg of D-Cy5 immediately post-induction (n = 1), 1 day (n = 1) or 2 days (n = 1) post-induction. The other six animals were injected with three doses of 20mg/kg of D-Cy5 immediately post-induction and both 1 day and 2 days post-induction. Animals were euthanized and tissues analyzed at varying times post-administration: 1 day (n = 1), 2 days (n = 1), 3 days (n = 1), 1 week (n = 2), 2 weeks (n = 2), or 4 weeks (n = 2) post-injection (Table 2).

**Table 2:** Protocol for intravenous injections of D-Cy5 after induction.

Animal #	Eye Induced	When injected*	When euthanized**
134	Right	Immediately	1 day
135	Right	5 hours	2 days
136	Right	Immediately	3 days
180	Right	Immediately, 1 day, 2 days	1 week
181	Right	5 hours, 1 day, 2 days	1 week
182	Right	5 hours, 1 day, 2 days	2 weeks
183	Right	5 hours, 1 day, 2 days	2 weeks
184	Right	5 hours, 1 day, 2 days	4 weeks
185	Left	5 hours, 1 day, 2 days	4 weeks
Primate	Right	3 days	6 days

*Post-induction

**Post-induction

For the primate targeting study, a single IV dose of 128mg D-Cy5 in 6.4 ml of sterile phosphate-buffered saline (PBS, 20mg/ml) was infused over 6 min, 3 days after induction.

### Tissue Collection

For rat tissue, animals were anesthetized to the deep surgical plane using ketamine/xylazine (80mg/5mg/kg). Animals were then euthanized by intracardiac perfusion of 4% paraformaldehyde-phosphate buffered saline (PF-PBS). Eyes were enucleated along with 5mm of adjacent ON. ONs were cryopreserved in 30% sucrose, embedded in optimal cutting tissue freezing medium and frozen-sectioned into 10μm-thick specimens. The retinas and ONs then were assessed histologically and histochemically.

For the primate experiment, tissues were collected 6 days post-induction (3d post-administration). The animal was initially anesthetized using ketamine/xylazine (10mg/2mg/kg), and then intubated and ventilated with 3.5% isoflurane to the deep surgical plane. The animal was then exsanguinated using normal saline via intracardiac puncture, followed after cardiac cessation by PF-PBS perfusion. The eyes were enucleated along with at least 1cm of optic nerve, and post-fixed in PF-PBS. The lamina was longitudinally hemisected, the distal ON cross-sectioned, and paraffin-embedded and cut at either 7μm or 25μm thickness. Sections were stained with DAPI and examined using confocal microscopy. D-Cy5 uptake was evaluated in retina, lamina, distal ON and in the white and gray matter of the brain (corpus callosum and parietal cortex).

### Immunohistochemistry

PF-PBS post-fixed retina, lamina and ON frozen sections were analyzed for fluorescent dendrimer signal (D-Cy5), as well as for RGCs (goat anti human Brn3a; Santa Cruz Sc-31984; 1:500 concentration), total inflammatory cells (mouse anti-human IBA1; Millipore MABN92; 1:500 concentration), and astrocytes (glial acidic fibrillary protein [rabbit anti-pig GFAP; Sigma-Aldrich clone GA5; 1:1000 concentration] and aldolase dehydrogenase 1L1 [rabbit anti-mouse Aldh1L1; Abcam ab87117]; 1:1000 concentration). We performed antigen recovery using citrate buffer. Primary antibodies made in different species (mouse antihuman IBA1 and rabbit antimouse Aldh1L1) enabled us to colocalize both Aldh1L1 and IBA1 without difficulty, using fluorescently labeled donkey anti-rabbit (Cy2) and donkey anti-mouse (Cy3) secondary antibodies. Histological analysis was performed using a 4-channel confocal microscope (Olympus E900).

## Results and Discussion

### Intravitreal Targeting Studies (rNAION model)

**Fig 1:**
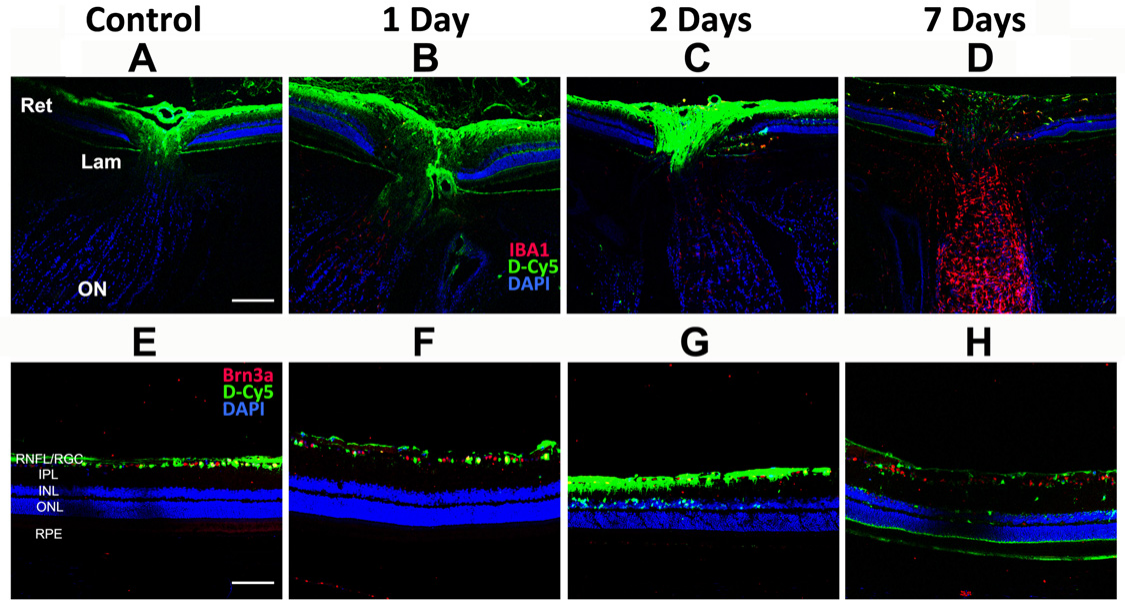
Intraocular distribution of intravitreally injected Cy5-labeled dendrimers (D-Cy5, green) in rNAION and control eyes. 60μg of D-Cy5 were injected intravitreally into either control or rNAION-induced eyes as described in the Methods section and Table 1. Animals were euthanized, and the posterior portion of the eye, including retina and first portion of the optic nerve (A-D) were examined, along with isolated retina (E-H), and analyzed for dendrimer uptake (in green) at 1, 2 or 7 days post-injection. Minimal signal is present in the laminar and retrolaminar ON in either control or rNAION-induced eyes at any time point; however, there is marked signal in the optic disc and inner layers of the peripapillary retina in the control eye and at both 1- and 2- days in the rNAION-induced eyes. By 7 days post-injection, there is only faint signal in the optic disc and peripapillary retina of the rNAION-induced eyes. In all panels, Green = D-Cy5 and Blue = DAPI. Red varies with figure as noted on the figure. A-D: Red = IBA1. E-H: Red = Brn3a. Scale bar (optic nerve): 200μm. Scale bar (retina): 100μm.

We analyzed uptake of IVT-injected D-Cy5 in both rNAION and non-induced (i.e., control) eyes. One day post-injection, D-Cy5 signal (green) was minimally detectable in the retina of the control (non-induced) eyes, primarily in the inner retinal layers (see Fig 1). There also was faint signal in the most anterior portion of the ON in the peripapillary region of these eyes (Fig 1A). Because of lack of ON signal seen 7d post-IVT injection (Fig 1D), we did not evaluate ON signal at later times in naïve animals.

One day following rNAION induction, D-Cy5 signal also was present in the inner retina and anterior ON (Fig 1, second column), similar to the control eye. There was increased retinal uptake of labeled dendrimer in the inner retinal layers at 2 days post-induction (Fig 1G). There was also increased signal in the intraocular portion of the ON at 2 days (Fig 1C) but this signal did not completely overlap with the ischemic region, which extends into the lamina and anterior optic nerve. By 7 days post-induction, most of the intraretinal D-Cy5 signal had disappeared, although there was some accumulation in a few IBA1(+) intraretinal macrophages (red). There was no detectable dendrimer signal in the anterior ON at this time.

### Single-Dose Systemic Targeting Studies (rNAION model)

**Fig 2:**
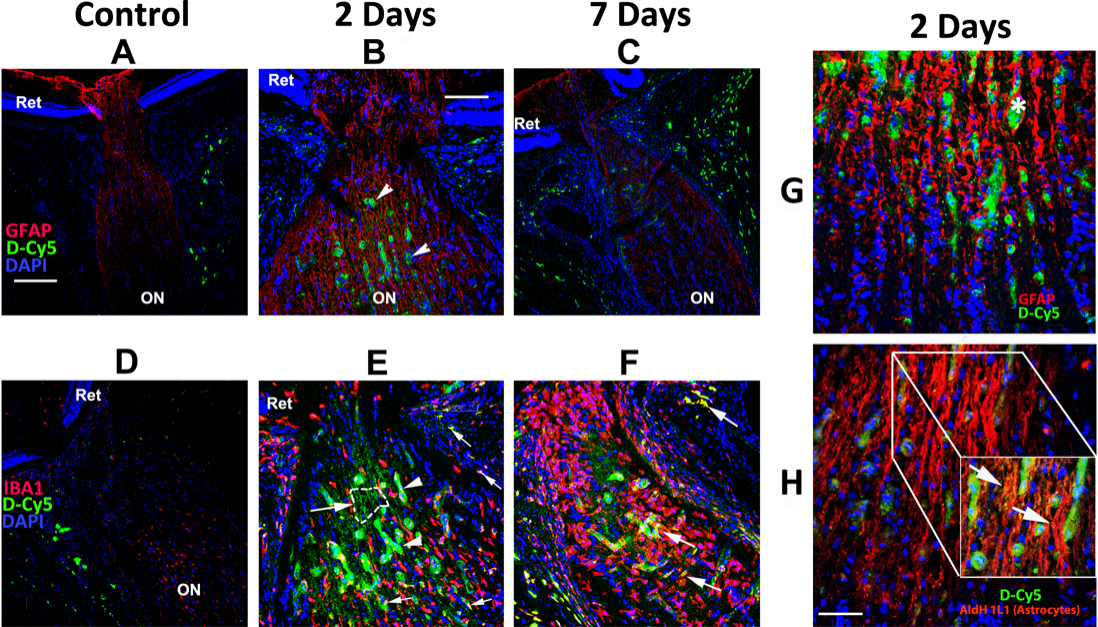
Fluorescent dendrimer distribution in the laminar region and distal optic nerve following rNAION induction and intravenous injection of Cy5-labeled dendrimers (D-Cy5). Cy5-labeled dendrimers were administered intravenously to animals in which rNAION was induced in one eye as indicated in the Methods section and Table 2. Animals were euthanized and optic nerves (ON) analyzed for dendrimer uptake (in green) in control animals and in rNAION animals 2 or 7 days post-induction. Confocal analysis reveals no accumulation of dendrimer in retinas or ONs of non-induced eyes at any timepoint, except in the ON sheath. Substantial dendrimer signal is seen in the ischemic region in rNAION-induced eyes at 2 days (B,E) and 7 days (C,F). G and H: Confocal analysis of astrocyte-associated dendrimer uptake at 2 days post-induction. G: GFAP (astrocyte structural protein)/Cy5 dendrimer colocalization. Dendrimer signal is found in both swollen sac-like structures associated with capillaries (asterisk) and in a linear pattern (arrows in B). H: Aldh1L1 (astrocyte-specific cytoplasmic protein)/dendrimer co-localization. There is extensive dendrimer signal overlap with the astrocyte cytoplasm (arrows in inset in H). There is also slight signal in ON sheaths of both non-induced and rNAION-induced eyes, presumably in macrophages. A-C and G, Red = GFAP. D-F, Red = IBA1. H, Red = Aldh1L1. Scale bars: A,C,D: 200μm. B,E,F: 100μm. Scale bar in G,H: 50μm. Scale bar in inset: 20μm.

A single dose of D-Cy5 administered intravenously immediately after unilateral rNAION induction did not accumulate in either the retinas or ONs of the non-induced eyes collected 1 day, 2 days or 3 days post-injection nor in the retinas at 1 week, 2 weeks, or 4 weeks post-injection (contralateral eye data for 2 days shown in Fig 2A and 2D). Dendrimer signal (green) was detectable in IBA1(+) circulating inflammatory cells (red) present within the optic nerve sheath and vascular plexus surrounding the nerve. Likewise, D-Cy5 was undetectable in the anterior ON in tissues collected 1d post-induction (Fig 2D), although dendrimer signal was present in the tissues outside the ON (Fig 2D). No dendrimer signal was detectable in either the retina or ON of animals injected immediately post-rNAION induction (data not shown). However, dendrimer signal was present at 2 days post-induction in a few invading macrophages, in dilated capillaries in the region of the ON lamina (Fig 2B and 2E, arrowheads), and surrounding the ON (Fig 2B) in the induced eye. D-Cy5 signal also was detected outside the capillaries in IBA1(+) cells 1d post-injection (Fig 2E, small arrows), accumulating in fibrillary structures consistent with astrocyte processes (Fig 2B, small arrows and 2E, dashed area indicated by an arrow). Although high-magnification images revealed little co-localization with the astrocyte structural protein GFAP (Fig 2G), dendrimer signal colocalized with Aldh1L1, an astrocyte-specific cytoplasmic protein (Fig 2E, and inset, Fig 2H)[22]. This indicates that, similar to lesions in the CNS, activated astrocytes accumulate dendrimers. Importantly, tissues from animals intravenously injected with D-Cy5 2 days post-induction and collected the next day (i.e., 3 days after rNAION induction and 1 day after systemic injection) also revealed dendrimer-loaded IBA1(+) macrophages accumulating in the laminar region/anterior ON, and in the ON sheath surrounding the ON (arrows, Fig 2F).

Minimal dendrimer signal was present in retinas of animals in which D-Cy5 was intravenously administered, regardless of whether the retinas were from eyes in which rNAION had been induced or were from contralateral (control) eyes (data not shown). Minimal signal was present in both microglia and astrocytes of the ON far distal to the primary stroke region (data not shown). No D-Cy5 was detectable in either the anterior or the posterior ON in tissues isolated for stereology (30d post-induction) when D-Cy5 was administered systemically 21 days post-induction (data not shown). Thus, activated macrophages and astrocytes within the primary ischemic region of the ON actively accumulated intravenously injected dendrimers only during the initial period of ON swelling and ischemia, suggesting that dendrimers only actively penetrate the lesion during blood brain barrier (BBB) disruption. This accumulation within the region of ON ischemia occurred only following systemic administration.

### Multiple-dose Systemic Targeting Study (rNAION model)

**Fig 3:**
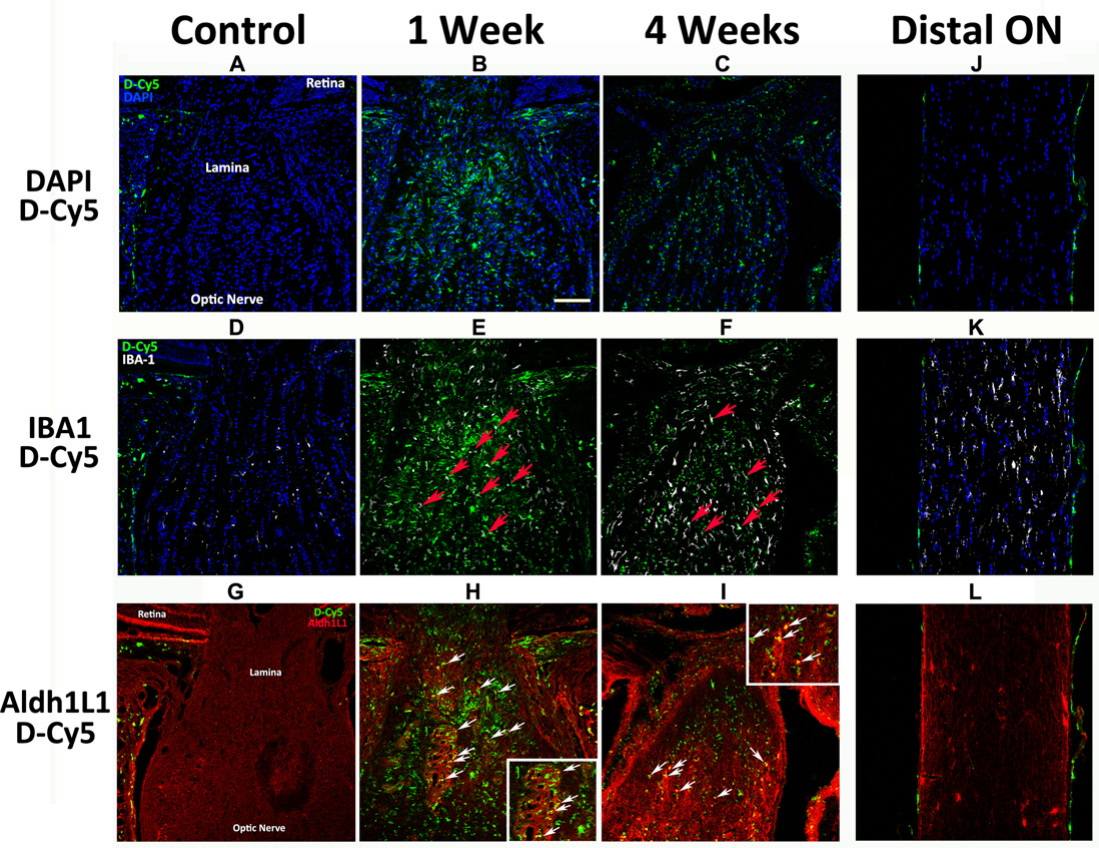
Confocal analysis of the time course of D-Cy5 in the cells of the lamina and optic nerve in rNAION and control eyes. A-I: sections of the retina:optic nerve junction region in naïve (A,D,G), 1 week post-rNAION induction (B,E,H), and 4 weeks post-induction (C,F,I) eyes. J-L: D-Cy5 localization in the distal optic nerve of an animal 1week post-rNAION induction. A,B,C. DAPI/D-Cy5 colocalization. Labeled D-Cy5 is external to the lamina in the naïve/non-induced eye (A) but accumulates at the primary rNAION lesion site (B). D-Cy5 is still present at 4 weeks post-induction (C), but the overall signal, although present, is less intense and less extensive. No D-Cy5 signal is present within the distal ON 1 week post-rNAION induction (J). D,E,F. D-Cy5/IBA1 co-localization within resident and invading inflammatory cells. There is no dendrimer signal co-localization with microglia in the lamina or the ON of the naïve eye 1 week post-injection (D). D-Cy5 colocalizes with IBA1(+) cells in the lamina and anterior ON of rNAION-induced eyes at both 1 week (E; red arrows) and 4 weeks post- induction (F; red arrows). G,H,I. Analysis of D-Cy5 and Aldh1L1 (astrocyte cytoplasm). G. There is no co-localization in the non-induced eye. H. D-Cy5 /Aldh1L1 co-localization is present 1 week post-induction (inset: higher magnification of same region). I. D-Cy5/Aldh1L1 continues to co-localize in the lamina region/anterior ON 4 weeks post-induction, but at a reduced level (inset:higher magnification of same region. There is minimal D-Cy5 present in the distal ON 1 week post-induction (J) and no co-localization of D-Cy5 with either IBA1(+) microglia (K) or Aldh1L1(+) astrocytes (L) at this time. Scale bar in B: 100μm.

**Fig 4:**
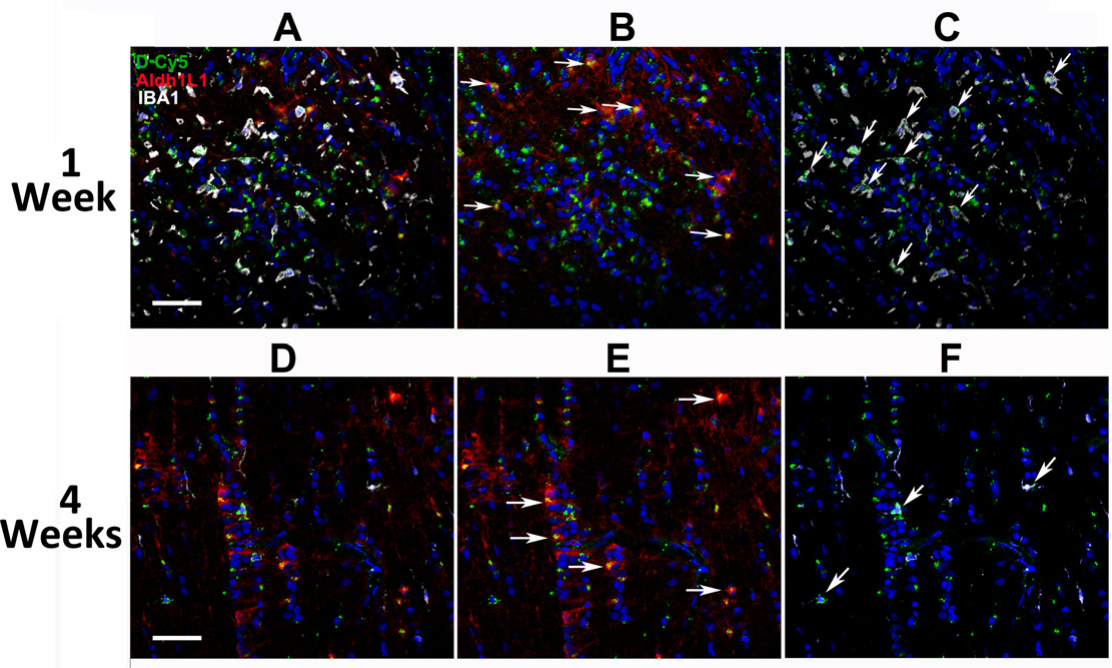
D-Cy5 persists in astrocytes and macrophages in the lamina after rNAION. Higher magnification (20X) confocal analysis of the same regions seen in Fig 3 at 1 (A-C) and 4 (D-F) weeks. A. Three-channel (astrocyte, inflammatory cell, DAPI nuclear stain) confocal photo of D-Cy5 localization in the rat lamina 1week post-induction. Strong D-Cy5 signal is scattered within the lamina. B. Dual-channel photo showing D-Cy5 localization at high levels in astrocyte cytoplasm (arrows) in the lesioned area, revealed by Aldh1L1 immunostaining. C. Dual-channel photo showing D-Cy5 localization in inflammatory cells (arrows). D. Three-channel confocal photo of D-Cy5 localization in the rat lamina 4 weeks post-induction. D-Cy5 is reduced overall in the lesion area. E. Identification of astrocyte-specific D-Cy5 co-localization. D-Cy5 is still concentrated in astrocytes (arrows) but at considerably lower signal levels than at one week. F. Dual-channel (inflammatory cell-specific) D-Cy5 co-localization. D-Cy5 is still present in IBA1(+) cells (arrows) but at lower signal levels than at 1 week. Scale bars: 100μm.

Relative fluorescent dendrimer signal was compared among animals administered three sequential doses of Cy5-dendrimer (5hr, 1d, 2d) and euthanized at 1, 2 or 4 weeks, and evaluated both at relatively low magnification to evaluate overall pattern of dendrimer accumulation (Fig 3), and at high magnification to identify accumulation in specific cell types (Fig 4). Systemically administered dendrimer was minimally present, if at all, in the lamina and anterior ON region of non-induced eyes (Fig 3A, 3D and 3G), in the entire ON of naïve eyes (data not shown) and in the ON distal to the rNAION lesion 1 week post-induction (Fig 3J–3L). One week post-induction, however, strong D-Cy5 was localized to the lamina and anterior ON (Fig 3B). This signal appeared to co-localize with both IBA1(+) cells (red arrows, Fig 3E) and astrocytes (red arrows, Fig 3H; inset shows higher magnification). Laminar localization continued in eyes examined 4 weeks post-induction (Fig 3C, 3F and 3I), although there was less D-Cy5 present at the later time point. Again, accumulation was detectable in IBA1 inflammatory cells (red arrows, Fig 3F), and astrocytes (Fig 3I; inset shows higher magnification). Thus, D-Cy5 localizes to and concentrates in the primary rNAION-associated ischemic lesion in the lamina and anterior ON within inflammatory cells and astrocytes, remaining present at least 1 month post-administration.

We further characterized the extent and cellular identity of localized D-Cy5 at extended times post-induction, by evaluating the co-localization of D-Cy5 within identical laminar lesion sites at 1- and 4-weeks (Fig 4). One week post-induction, strong D-Cy5 signal co-localized in the lamina (Fig 4A) in both Aldh1L1 (+) astrocyte cytoplasm (arrows, Fig 4B) and IBA1(+) inflammatory cells (arrows, Fig 4C), revealing sustained accumulation in both laminar cellular components. D-Cy5 signal was still strong 4 weeks post-induction in the astrocyte compartment (arrows, Fig 4F) as well as in the macrophage compartment, suggesting that systemic dendrimer administration is appropriate for sustained therapy to both cell species after ischemia.

### Single-Dose Systemic Targeting Study (pNAION Model)

**Fig 5:**
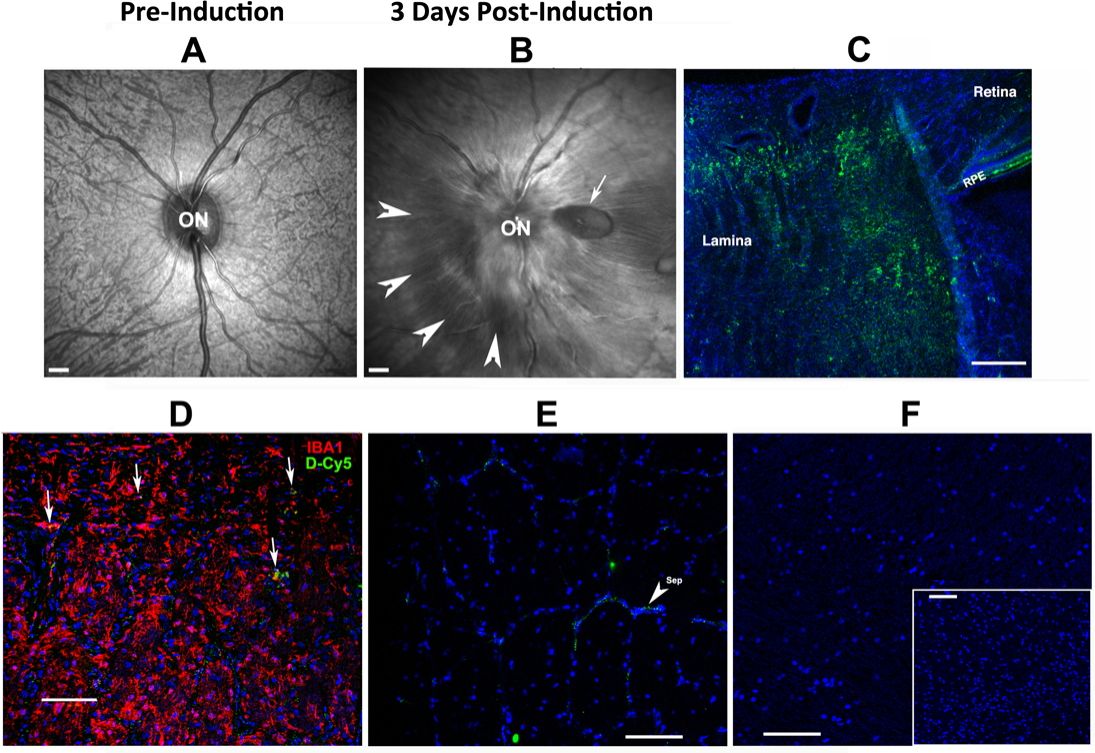
D-Cy5 is present in the region of optic nerve ischemia in eyes with pNAION but not in white or gray matter in the brain. A. SD-OCT baseline scan of the primate optic disk and surrounding retina (*en face* view). The optic disk (ON) and retinal nerve fiber layer entering the nerve are flat against the back of the eye. The underlying choroidal vasculature is visible through the retina. B. SD-OCT *en face* scan of the posterior portion of the same eye 3 days post-pNAION induction. The disk (ON) is grossly swollen, with blurring of the disk margin. There is a serous peripapillary retinal detachment (arrowheads), and a peripapillary retinal nerve fiber layer hemorrhage (arrow). C-F: Histology post-pNAION of the same animal 3d post-D-Cy5 injection (6d post-induction). C. Laminar region, longitudinal section. D-Cy5 is concentrated in the laminar region to a depth of 1mm (approximately the entire laminar thickness). D-Cy5 is also present in the RPE. D. Lamina, IBA1 immunohistochemistry. D-Cy5 co-localizes in individual laminar inflammatory cells (arrows) that are concentrated in the pNAION lesion. E. Distal ON. There is minimal D-Cy5 signal that is restricted to small areas of optic nerve connective-tissue septae (sep; arrowhead). F. CNS white and gray matter. There is minimal D-Cy5 signal in both white (corpus callosum) and gray (inset: cortex) matter. Scale bars in A and B: 500μm, Scale bar in C: 200μm. Scale bars in D,E and F: 100μm. Scale bar in F (inset): 100μm.

Because intravitreally administered D-Cy5 did not penetrate through to the (relatively small) whole rodent laminar region whereas systemic dendrimers did, we elected to administer D-Cy5 intravenously following induction of pNAION in a single adult male rhesus monkey. The induction generated a moderately severe lesion associated with disk swelling, peripapillary hemorrhages, and a localized peripapillary serous retinal detachment (Fig 5B; compare with baseline, Fig 5A). The primate SD-OCT findings were consistent with the severity of pNAION induction that, like human NAION, can result in localized serous retinal detachment and disk hemorrhages.

Following administration of a single IV dose (20mg/kg) of D-Cy_5_ (see Methods), urinary excretion was rapid, with blue urine appearing within 10 minutes of injection (data not shown). Fixed tissues revealed accumulation of D-Cy5 in the pNAION-associated lesion in the lamina region (Fig 5C, lamina) as well as in the retinal pigment epithelium surrounding the lamina (Fig 5C; RPE). Immunohistochemistry revealed D-Cy5 colocalization within individual laminar microglia/macrophages (Fig 5D, arrows). Minimal D-Cy5 signal was detectable in the distal ON (ON 5mm distant from the pNAION lesion)(Fig 5E). D-Cy5 signal was undetectable in other distant CNS tissues, either brain white matter (corpus callosum: Fig 5F) or cerebral cortex (inset, Fig 5F). Thus, D-Cy5 concentrates in the laminar region of both the rNAION and the pNAION models, without crossing the undamaged BBB into either the distal ON or CNS, and persists for days, if not weeks.

The current study indicates that intravenously injected 4^th^ generation PAMAM dendrimer nanoparticles target the area of optic nerve ischemia and inflammation, with minimal uptake in the healthy eyes, in both the murine and primate models of NAION. Thus, this method may be an effective therapeutic targeting strategy not only for human NAION but for other conditions that damage the optic nerve[17,18].

In contrast with the distribution of intravenously injected dendrimers within the optic nerve, the ocular distribution of intravitreally injected D-Cy5 after induction of rNAION indicates that this route of administration provides minimal access of dendrimers to the anterior ON and ischemic region despite the prolonged time period that dendrimers appear to remain in the vitreous (~2 days). IVT administration of D-Cy5 results in accumulation in the inner retina 1 day post-injection both in eyes with rNAION and in control eyes (see Fig 2E and 2F), with the pattern of accumulation over time (>2d) being consistent with uptake into Muller and retinal microglia cells—cells that also are activated after induction of rNAION[9]. Seven days post-induction, D-Cy5 was even present in the RPE. Although IVT administration of dendrimer-linked compounds is unlikely to be useful in the treatment of primary optic nerve disease, our data, as well as that of others[20,23], suggest that medication linked to PAMAM dendrimers administered by IVT injection may be useful in targeting diseases where activated microglia, Muller cells and RPE are involved, such as retinal and subretinal diseases, as well as the intraocular portions of the ON axons[23].

Systemic administration of D-Cy5, unlike IVT dosing, effectively targeted the entire primary rNAION and pNAION lesions when injected at early times (1–3 days) post-induction. D-Cy5 accumulated in both macrophages and activated astrocytes in the anterior ON (see Figs 3 and 5) and in activated (extrinsic) macrophages in the ON surround, remaining present even 30 days post-injection; however, no D-Cy5 was detectable in either the anterior or distal ON when D-Cy5 was systemically injected 3 weeks post-induction (data not shown). These results suggest that effective dendrimer penetration into the ON only occurs when there is disruption of the BBB, at which time the dendrimers are selectively taken up by both invading macrophages and activated/reactive astrocytes. There are also important cellular differences in systemically administered dendrimer uptake in the ischemic retina, compared with ON tissue. Although retinal inflammation is associated with Muller cells in degenerating retinas[24], D-Cy5 does not accumulate in these cells in a retinal ischemia-reperfusion model[20,25]. This is different from the resident astrocyte response to ON ischemia. In our study, following isolated ON ischemia, D-Cy5 accumulated in a pattern similar to that seen in a model of cerebral ischemia[16]. It is unknown if dendrimers are concentrated only in cells already present at the lesion site or if they also are brought in by activated systemic extrinsic macrophages that also invade the lesion post-induction. Regardless, we believe that dendrimer compounds are likely to be effective in the treatment of ON disease only when there is acute damage or in circumstances resulting in extrinsic macrophage invasion as is the case after acute CNS ischemia and in the early stages of human NAION. Thus, dendrimers may be extremely useful as a targeting treatment modality in NAION, enabling selective dendrimer uptake within the damage site and improving drug efficacy while reducing side effects.

The differential biodistribution of PAMAM dendrimers in eyes with experimental NAION after both local (IVT) and systemic (IV) administration offers new insights into the role of BBB impairment and dendrimer transport in the eye. Although both methods may be effective for delivering drugs to damaged retina, systemic administration appears to result in superior uptake and retention in eyes with optic nerve injury, at least of the type produced in our animal models of NAION.

## Conclusions

The current report provides the data needed to design a study for NAION-treatment using dendrimer-linked neuroprotective agents. We have done this by defining the ability of dendrimer nanoparticles to target the NAION lesion, the route of most effective application for post-ischemic treatment, the time window of administration, cellular localization of the fluorescent nanoparticles and by determining that the dendrimer-nanoparticle approach is potentially appropriate to treat ischemic neuropathy in rodents as well as primates. PAMAM dendrimers may provide an exciting new approach to NAION therapy. Dendrimer-drug combinations should enable effective targeting, high intracellular concentration, and sustained release of the associated drug, enhancing the overall effect of many drugs without a corresponding increase in systemic toxicity.
